# Enhanced Superpixel-Guided ResNet Framework with Optimized Deep-Weighted Averaging-Based Feature Fusion for Lung Cancer Detection in Histopathological Images

**DOI:** 10.3390/diagnostics15070805

**Published:** 2025-03-21

**Authors:** Karthikeyan Shanmugam, Harikumar Rajaguru

**Affiliations:** Bannari Amman Institute of Technology, Tamil Nadu 638401, India; karthikeyan@bitsathy.ac.in

**Keywords:** SLIC segmentation, RN-X, PSO, RDO, MLP, cross-validation

## Abstract

**Background/Objectives:** Lung cancer is a leading cause of cancer-related mortalities, with early diagnosis crucial for survival. While biopsy is the gold standard, manual histopathological analysis is time-consuming. This research enhances lung cancer diagnosis through deep learning-based feature extraction, fusion, optimization, and classification for improved accuracy and efficiency. **Methods:** The study begins with image preprocessing using an adaptive fuzzy filter, followed by segmentation with a modified simple linear iterative clustering (SLIC) algorithm. The segmented images are input into deep learning architectures, specifically ResNet-50 (RN-50), ResNet-101 (RN-101), and ResNet-152 (RN-152), for feature extraction. The extracted features are fused using a deep-weighted averaging-based feature fusion (DWAFF) technique, producing ResNet-X (RN-X)-fused features. To further refine these features, particle swarm optimization (PSO) and red deer optimization (RDO) techniques are employed within the selective feature pooling layer. The optimized features are classified using various machine learning classifiers, including support vector machine (SVM), decision tree (DT), random forest (RF), K-nearest neighbor (KNN), SoftMax discriminant classifier (SDC), Bayesian linear discriminant analysis classifier (BLDC), and multilayer perceptron (MLP). A performance evaluation is performed using K-fold cross-validation with K values of 2, 4, 5, 8, and 10. **Results:** The proposed DWAFF technique, combined with feature selection using RDO and classification with MLP, achieved the highest classification accuracy of 98.68% when using K = 10 for cross-validation. The RN-X features demonstrated superior performance compared to individual ResNet variants, and the integration of segmentation and optimization significantly enhanced classification accuracy. **Conclusions:** The proposed methodology automates lung cancer classification using deep learning, feature fusion, optimization, and advanced classification techniques. Segmentation and feature selection enhance performance, improving diagnostic accuracy. Future work may explore further optimizations and hybrid models.

## 1. Introduction

Cancer is a complex set of diseases marked by uncontrolled growth and spread [1], unlike benign tumors, which remain localized, whereas malignant tumors invade and damage nearby tissues. Lung cancer is the leading type in men and the third in women, closely linked to smoking, and it is the primary contributor to cancer-associated mortality globally [2]. The WHO projected that cancer would become the top global cause of death by 2020 [3], with lung cancer alone causing around 1.80 million deaths. Projections indicate that, by 2035, lung cancer might contribute up to 60% of all cancer-related fatalities [4]. Early-stage cancers that are operable have a 5-year survival rate of approximately 34%, but for inoperable cases, the rate drops to under 10%. Lung cancer, which is predominantly classified into non-small-cell lung carcinoma (NSCLC) and small-cell lung carcinoma (SCLC) [5], shows varying characteristics. NSCLC, making up about 85% of cases, includes adenocarcinoma (ADC), squamous cell carcinoma (SCC), and large-cell carcinoma (LCC). The remaining 15% are SCLC cases.

Histopathological examination identifies lung cancer subtypes through biopsy reports [6], crucial for accurate diagnosis and effective treatment planning [7]. Computer-aided diagnosis (CAD) systems support pathologists by providing automated assessments to prevent misclassification [8]. Advances in artificial intelligence (AI) have enhanced both the precision and effectiveness of histopathological slide analysis. This study centers on categorizing lung cancer biopsy images into two distinct categories, adenocarcinoma and benign using deep learning frameworks.

### Contribution of the Work

The major contribution of this research work can be summarized as follows:

1. Histopathological images are preprocessed using an adaptive fuzzy filter and segmented using the modified SLIC algorithm.
2. The segmented images are passed through deep learning models such as ResNet-50, ResNet-101, and ResNet-152 for feature extraction, followed by a proposed deep-weighted averaging feature fusion technique to generate RN-X features.
3. The extracted features from the ResNet models and RN-X are put into a selective feature pooling layer, which leverages PSO and RDO optimization algorithms for feature selection.
4. Finally, the classification layer implements the classifiers such as SVM, DT, RF, KNN, SDC, BLDC, and MLP, evaluated using K-fold cross-validation with K values of 2, 4, 5, 8, and 10.

This study is organized as follows: Section 2 provides a review of recent research on lung cancer detection. Section 3 presents the methodology. Section 4 details the proposed deep-weighted averaging feature fusion technique and discusses the selective feature pooling layer, incorporating PSO and RDO methods, along with the classification layer. Section 5 focuses on result comparisons. Finally, Section 6 highlights key findings and suggests directions for future research.

## 2. Review of Lung Cancer Detection

Over recent decades, various approaches have been proposed for the automated detection, segmentation, and classification of histopathological images using machine learning (ML) and deep learning (DL). Anthimopoulos et al. [9] developed a Convolutional Neural Network (CNN) with five convolutional layers using Leaky ReLU activation, average pooling, and three fully connected layers. Lizuka et al. [10] combined Inception v3 and an RNN to classify stomach and colon biopsies, incorporating regularization and augmentation for robustness. Wang et al. [11] used a CNN for lung cancer pathology, achieving 90.1% accuracy with Softmax activation. Gessert et al. [12] explored multiresolution EfficientNet for skin sore classification. Liu et al. [13] applied wavelet-based denoising to address noise in histopathological images, achieving 94.37% accuracy on the BreakHis dataset.

Zhou et al. [14] designed a hierarchical model using SVM and SURF features, achieving 91.14% accuracy, but performance at 400× magnification needed improvement. Wang et al. [15] introduced FE-BkCapsNet, combining CNNs with CapsNet and yielding up to 94.52% accuracy. Aresta et al. [16] used DenseNet121, achieving 87% accuracy on the BACH 2018 dataset. Spanhol et al. [17] combined CNN predictions, achieving 84% accuracy on BreakHis dataset at 200X magnification, while Filipczuk et al. [18] focused on nuclei segmentation and trained several classifiers using 25 shape and texture features.

Nada Mobarak et al. [19] created CoroNet, a CNN based on Xception, achieving 88.67% accuracy for breast cancer detection on the CBIS-DDSM dataset. Teresa et al. [20] applied CNN models to the Bioimaging 2015 dataset, segmenting images into 512 × 512-pixel patches and achieving up to 83.3% accuracy. Ahsan Rafiq et al. [21] proposed a three-CNN model for breast cancer classification, achieving 90.10% accuracy. Hameed et al. [22] fine-tuned Visual Geometry Group (VGG) models and used an ensemble approach, outperforming individual models. Wang et al. [23] achieved 96.19% accuracy using wavelet transforms and SVM with a genetic algorithm for feature selection.

## 3. Materials and Methods

**Figure 1:**
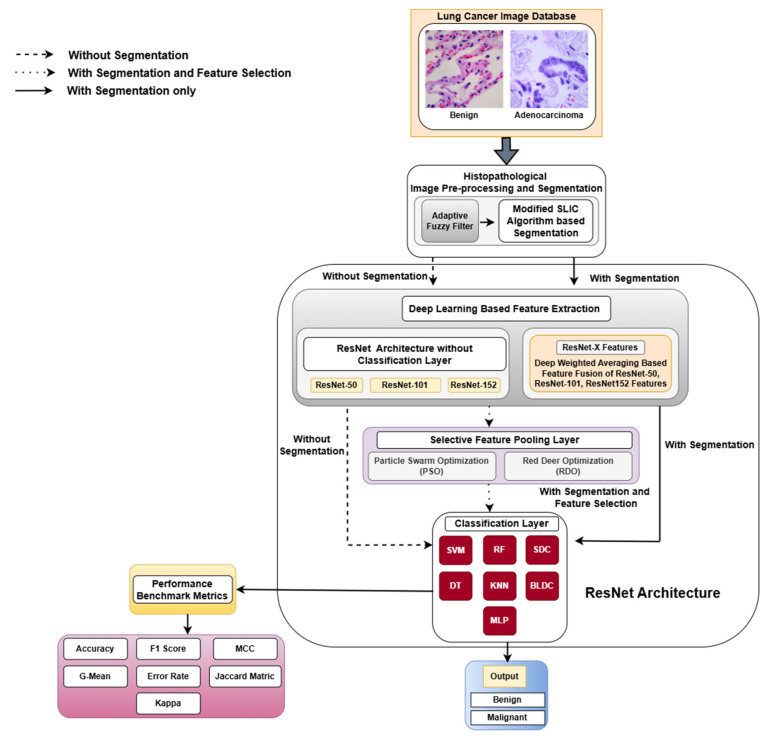
Detailed workflow of the detection of lung cancer abnormalities. MCC—Matthews Correlation Coefficient.

This section offers an in-depth overview of the resources and methodologies employed in the classification of lung and colon cancers. The methodological framework of this study is illustrated in Figure 1.

### 3.1. Dataset Used

The LC25000 dataset, introduced in 2020 [24], contains 25,000 color images of five tissue types, expanded through augmentation from an original 1250 images of cancer. The images were resized to 768 × 768 pixels and verified for Health Insurance Portability and Accountability Act (HIPAA) compliance. This study focuses on 5000 benign and 5000 adenocarcinoma lung cancer images. Adenocarcinoma originates in glandular cells and often spreads to the alveoli. Benign tissues, while non-cancerous, typically require surgical removal and biopsy to confirm their nature.

### 3.2. Image Preprocessing

Histopathological image analysis is crucial for assessing tumor characteristics, clinical staging, and predicting patient survival [25]. However, these images face challenges such as complex geometric patterns and textures, critical textural features, image dimension and resolution variations, and color and noise issues. This study demonstrates that applying an adaptive fuzzy filter to resized images (224 × 224) enhances clarity by reducing noise and artifacts, resulting in more accurate diagnoses. The filtered images are then used for segmentation of the region of interest (ROI).

### 3.3. Modified SLIC Algorithm-Based Segmentation

A superpixel groups adjacent pixels that exhibit similar color, luminance, and texture properties to segment an image [26]. The SLIC algorithm allocates M initial seed points uniformly throughout the image. For an image with N pixels segmented into M superpixels, each superpixel contains N/M pixels. The separation between neighboring seed points is $S=\sqrt{N/M}$. The feature vector of the centroid is $C_{i}={\left[l_{i},a_{i},b_{i},x_{i},y_{i}\right]}^{T}$, combining the CIELAB color values $\left[l_{i},a_{i},b_{i}\right]$ and the pixel position $\left(x_{i},y_{i}\right)$. To enhance segmentation, the SLIC algorithm adjusts each centroid to the point with the minimal gradient in a 3 × 3 neighborhood. After initialization, it iteratively clusters pixels by assigning them to the nearest center and computing distances within a 2S × 2S neighborhood of each center. In the SLIC algorithm, the measure of proximity between a candidate pixel and the centroid of a cluster is expressed as

(1) $$d_{ss}\left(i,j\right)=\sqrt{{\left(x_{i}-x_{j}\right)}^{2}+{\left(y_{i}-y_{j}\right)}^{2}}$$

(2) $$d_{cs}\left(i,j\right)=\sqrt{{\left(l_{i}-l_{j}\right)}^{2}{\left(a_{i}-a_{j}\right)}^{2}+{\left(b_{i}-b_{j}\right)}^{2}}$$

(3) $$d_{ts}\left(i,j\right)=\sqrt{d_{ss}^{2}+\alpha d_{cs}^{2}}$$

Here, i denotes the centroid label, and j denotes the pixel index in the 2S × 2S neighborhood. $d_{ss}$ represents spatial similarity, $d_{cs}$ represents color similarity, and $d_{ts}$ is the total similarity with a lower $d_{ts}$ signifying higher similarity. The parameter α=s/m, where s denotes the neighborhood size and m indicates the compact factor balancing $d_{ss}$ and $d_{cs}$, typically ranges from 1 to 40. This paper introduces a modified SLIC algorithm that simplifies calculations using a 3-dimensional feature vector consisting of spatial co-ordinates $\left(x,y\right)$ and grayscale feature (gs). The distance between a candidate pixel and the cluster centroid is revised as follows:

(4) $$d_{gs}\left(i,j\right)=\sqrt{{\left(g_{i}-g_{j}\right)}^{2}}$$

(5) $$d_{ts}^{\prime }\left(i,j\right)=\sqrt{d_{s}^{2}+\alpha d_{gs}^{2}}$$

where $d_{gs}$ denotes pixel similarity in grayscale values, and $d_{ts}^{\prime }$ represents the overall similarity between the cluster centroid and the pixel co-ordinates. The algorithm of the modified SLIC superpixel segmentation is as follows.

Step 01: The microscopic color cell image is initially transformed into a grayscale format. It is then randomly split into K segments. Given the grayscale probability distribution $p_{0},p_{1},...,p_{n-1}$, and multiple thresholds $t_{1},t_{2},...,t_{k}$ (where $t_{1}<t_{2}<...<t_{k}$), the entropy for these segments can be expressed as follows:

(6) $$\varphi \left(t_{1},t_{2},...,t_{k}\right)=\log\left(\sum_{i=0}^{t_{1}}p_{i}\right)+\log\left(\sum_{i=t_{1}+1}^{t_{2}}p_{i}\right)+...+\log\left(\sum_{i=t_{k}+1}^{n}p_{i}\right)\;-\frac{\sum_{i=0}^{t_{1}}p_{i}\log p_{i}}{\sum_{i=0}^{t_{1}}p_{i}}-\frac{\sum_{i=t_{1}+1}^{t_{2}}p_{i}\log p_{i}}{\sum_{i=t_{1}+1}^{t_{1}}p_{i}}-...-\frac{\sum_{i=s_{k}+1}^{n}p_{i}\log p_{i}}{\sum_{i=t_{k}+1}^{t_{1}}p_{i}}$$

The multiple thresholds $t_{1},t_{2},...,t_{k}$ for ideal classification for each segment adhere to the principle of maximum entropy, as follows:

(7) $$\left(t_{1},t_{2},...,t_{k}\right)=\arg\left\{\underset{t_{1},t_{2},...,t_{k}}{max}\left[\varphi \left(t_{1},t_{2},...,t_{k}\right)\right]\right\}$$

These thresholds are determined using a conditional iteration algorithm.

Step 02: Using the optimal thresholds, the grayscale image is divided into K+1 intervals: $\left[X_{i},X_{i+1}\right]$, $X_{i}\in \left\{t_{1},t_{2},...,t_{k}\right\}$, and $i\in \left\{1,2,...,k\right\}$. Each interval $\left[X_{i},X_{i+1}\right]$ is transformed into $\left[Y_{i},Y_{i+1}\right]$ with a contrast-enhancing function, $f\left(x\right)\circ f\left(x\right)$. The function is convex in $\left[X_{i},X_{m}\right]$ and concave in $\left[X_{m},X_{i+1}\right]$, with turning point $\left[X_{m},Y_{m}\right]$, where $Y_{m}=\left(Y_{i}+Y_{i+1}\right)/2$. $X_{m}$ is determined using the least squares principle:

(8) $$X_{m}=\frac{\int_{X_{i}}^{X_{i}}xp(x)dx}{\int_{X_{i}}^{X_{i}}p(x)dx}$$

To simplify image processing, grayscale transformation is modeled by the following function:

(9) $$f(x)=ax^{r}+b,x\geq 1,r\geq 1$$

Here, $a=\left(Y_{i+1}-Y_{i}\right)/\left(X_{i+1}^{r}-X_{i}^{r}\right),b=Y_{i}-aX_{i}^{r}$. Varying r generates different transformation curves. A higher r improves gray equalization in the interval $\left[X_{i},X_{i+1}\right]$. By choosing appropriate $Y_{i},\;i\in \left\{1,2,...,n\right\}$ and r values, regional balance and contrast can be enhanced, leading to a more evenly adjusted and contrasted image.

Step 03: initialize clustering centers $C_{i}$ using grid superpixels with side length $S=\sqrt{N/M}$, and assign labels.

Step 04: move each center, $C_{i}$, to the location with the minimum gradient within its 3 × 3 neighborhood.

Step 05: calculate the similarity distance, $d^{\prime }$, from each pixel, j, to $C_{i}$ within a radius, S, which matches the circular shape of the cell image.

Step 06: Set dist(i)=∞. If $d^{\prime }(i)<dist(i)$ and is within range, update $d^{\prime }(i)$ to dist(i), and assign the label i to pixel j.

Step 07: Repeat steps 4 to 6 until clustering converges. Recalculate each cluster’s mean grayscale and spatial features to update centers.

Step 08: merge isolated small superpixels using an adjacent merging strategy for improved fit and coherence.

**Figure 2:**
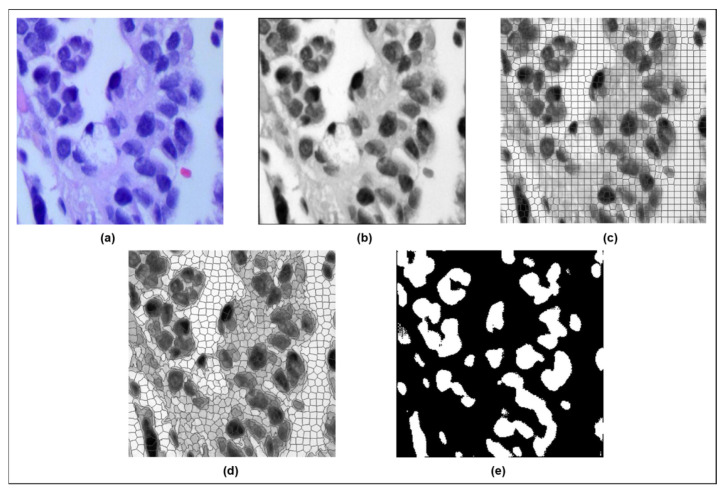
(**a**) Original ACA image; (**b**) adaptive fuzzy filtered ACA image; (**c**) original SLIC superpixel segmentation; (**d**) modified SLIC superpixel segmentation; (**e**) modified SLIC segmentation result for the adenocarcinoma class (ACA).

Figure 2 shows the image progression from the (a) original to the (b) filtered image, followed by (c) original SLIC superpixel segmentation, (d) modified SLIC superpixel segmentation, and finally, the (e) modified SLIC segmentation result for the adenocarcinoma class (ACA).

## 4. Deep Learning Architecture

**Figure 3:**
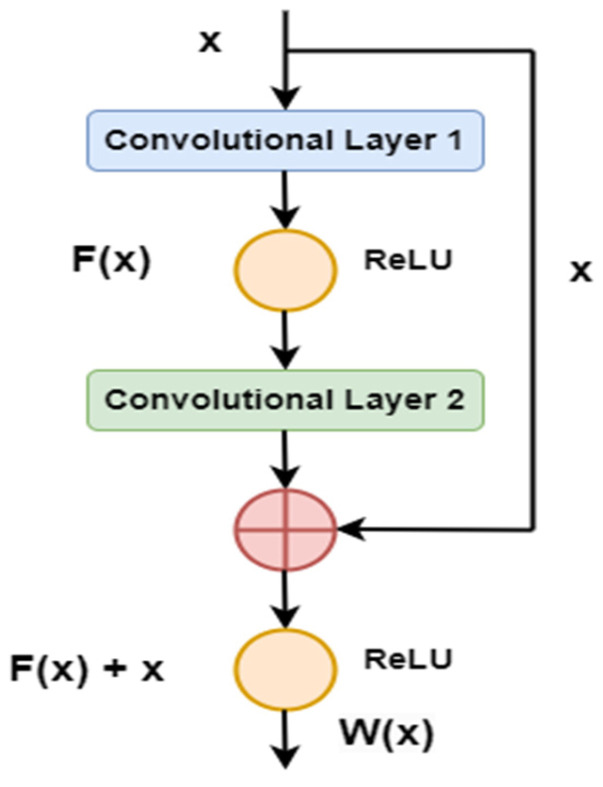
Residual mapping function.

Deep learning networks are powerful but face challenges like saturation, accuracy degradation, and vanishing or exploding gradients. Architectures like ResNet-50 (RN-50), ResNet-101 (RN-101), and ResNet-152 (RN-152) address these issues using residual learning and identity mapping [27]. These architectures use shortcut connections that help mitigate the vanishing gradient problem and prevent overfitting [28]. The mapping function, as shown in Figure 3, is expressed as follows:

(10) $$W\left(x\right)=F\left(x\right)+x$$

In Table 1, A, B, C, and D represent the number of blocks in the first, second, third, and fourth stages of the ResNet versions, respectively.

**Figure 4:**
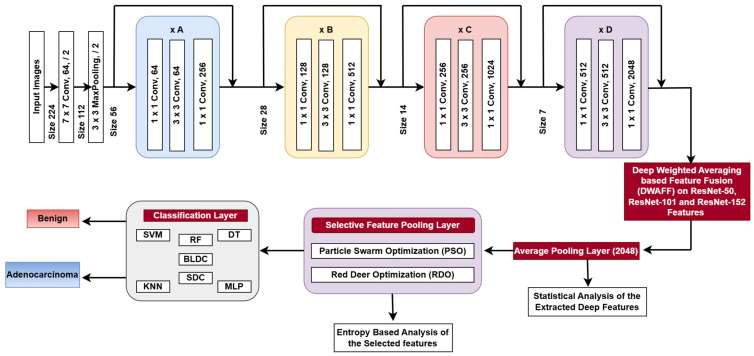
Proposed ResNet-X architecture.

The ResNet architecture configurations consist of different stages that are stacked across various ResNet versions, resulting in a 1D feature vector with 2048 elements for each image, as shown in Figure 4.

### 4.1. Proposed DWAFF Technique for ResNet-X Features

This study proposes a deep-weighted averaging-based feature fusion (DWAFF) technique. In this method, ResNet variants are evaluated, and weights are assigned to their feature vectors based on performance. By prioritizing contributions from each architecture, weights (ranging from 0 to 1) are adjusted in increments of 0.1 through trial and error. The final fused feature set for each image is computed using the weighted sum of the features as follows:

(11) $$ResNet\_X_{\mathrm{feature}}=w_{1}\times ResNet\_152_{\mathrm{feature}}+w_{2}\times ResNet\_101_{\mathrm{feature}\;}+w_{3}\times ResNet\_50_{\mathrm{feature}\;}$$

The optimal weight combination for feature fusion was determined using K-fold cross-validation on the dataset for each architecture—RN-50, RN-101, and RN-152—across various K values of 2, 4, 5, 8, and 10. Among these, ResNet 152 demonstrated the highest accuracy, followed by ResNet 101 and ResNet 50. The weight values for the architecture were chosen through a trial-and-error method, constrained to lie between 0 and 1, with their sum equal to 1. The best-performing combination was identified as 0.45 for RN-152 ($w_{1}$), 0.35 for RN-101 ($w_{2}$), and 0.20 for RN-50 ($w_{3}$). These weights were subsequently applied to fuse features using Equation 11. Additionally, the mean value of the normal class is added to the normal features, and the mean value of the abnormal class, is added to the abnormal features, enhancing class separation and improving classification. The equation for generating DWAFF-based RN-X features is given by the following.

For normal cases,

(12) $$ResNet\_X_{\mathrm{feature}\;\left(i,j\right)}=\frac{\left(w_{1}\times ResNet\_152_{\mathrm{feature}\;\left(i,j\right)}+w_{2}\times ResNet\_101_{\mathrm{feature}\;\left(i,j\right)}+w_{3}\times ResNet\_50_{\mathrm{feature}\;\left(i,j\right)}\right)}{3}+mean\_normal$$

For abnormal cases,

(13) $$ResNet\_X_{\mathrm{feature}\;\left(i,j\right)}=\frac{\left(w_{1}\times ResNet\_152_{\mathrm{feature}\;\left(i,j\right)}+w_{2}\times ResNet\_101_{\mathrm{feature}\;\left(i,j\right)}w_{3}\times ResNet\_50_{\mathrm{feature}\;\left(i,j\right)}\right)}{3}+mean\_abnormal$$

In this context, i ∈0 to 2047 denotes the features extracted per image, j represents the image index, j ∈0 to 4999 for the normal class and 5000 to 9999  for the abnormal class, and mean_normal is the average of mean values from all three ResNet variants for normal images, while mean_abnormal represents the same abnormal images. The Algorithm 1 for the proposed DWAFF technique for ResNet-X features is shown below.
**Algorithm 1. DWAFF Based Feature Fusion for ResNet-X Features****Step****Description****Details**Step 01Extract Features    Extract feature vectors for each image from ResNet-50, ResNet-101, and ResNet-152.    Store the feature vectors: ResNet-50_feature [i, j], ResNet-101_feature [i, j], and ResNet-152_feature [i, j], where i∈[0,2047] and j∈[0,9999].    Perform K-fold Cross-Validation (K values are 2, 4, 5, 8, 10).    Train the classifiers on the dataset set split.    Evaluate performance of the classifiers for the different values of K using performance metrics.Step 02Set Initial Weight Range    Initialize a range of possible weights, $w_{1}$, $w_{2},$ and $w_{3}$, based on the trial-and-error method, such that their sum must be equal to 1, based on the results obtained from K-fold cross-validation.Step 03Identify Optimal Weights    For each weight combination, calculate the average performance across the K-folds.    Select the weight combination that achieves the highest average performance.    Optimal weights are identified as, 0.45 for ResNet-152 ($w_{1}$), 0.35 for ResNet-101 ($w_{2}$), and 0.20 for ResNet-50 ($w_{3}$).Step 04Compute Mean Values    Compute mean_normal and mean_abnormal, across all three ResNet variants.Step 05Fuse Features for Final Feature Set    For normal cases (j∈[0.4999]): fuse features of normal cases using Equation (12).    For abnormal cases (j∈[5000,9999]): fuse features of abnormal cases using Equation (13).Step 06Output Final Fused Features    The final fused feature set for both normal and abnormal cases, which are ResNet-X features, are used for subsequent classification tasks.

#### Statistical Analysis

To enhance cancer classification accuracy with a reduced number of features, statistical measures play a crucial role in further analysis. The extracted features from ResNet-50, ResNet-101, ResNet-152, and the fused features from ResNet-X are analyzed by calculating statistical metrics such as the mean, variance, skewness, kurtosis, Pearson correlation coefficient (PCC), and canonical correlation analysis (CCA). These measures help assess how effectively the features capture lung cancer characteristics in both cancerous and non-cancerous data.

Table 2 presents the statistical parameters for the ResNet-50, ResNet-101, ResNet-152, and DWAFF-ResNet-X architectures for normal (N) and abnormal (ACA) cases. DWAFF-ResNet-X shows the best average performance with the highest mean values for both N (0.453891) and ACA (0.453709), outperforming the ResNet models, whose performance improves with depth. In terms of variance, DWAFF-ResNet-X has the lowest values (N: 0.380702; ACA: 0.444597), indicating more consistent performance compared to the higher variances in ResNet models, especially in ACA. For skewness, DWAFF-ResNet-X (N: 3.767961; ACA: 4.486885) shows a more symmetrical performance distribution, whereas ResNet models have higher skewness, indicating more inconsistent results. Kurtosis is also lower for DWAFF-ResNet-X (N: 21.14865; ACA: 33.4781), reflecting fewer extreme outliers than ResNet models, which have higher kurtosis values. PCC is highest in DWAFF-ResNet-X (N: 0.938638; ACA: 0.944338), indicating stronger alignment between predictions and outcomes compared to ResNet models. CCA also improves with model depth, with DWAFF-ResNet-X showing the highest CCA for ACA (0.8816). The dice coefficient values indicate the performance of the models in segmentation tasks. ResNet-50 shows moderate performance, with slightly better accuracy for normal cases. ResNet-101 outperforms ResNet-50, especially for normal cases. ResNet-152 demonstrates significant improvement, achieving higher accuracy for both normal and abnormal cases. DWAFF-ResNet-X delivers the best performance, with the highest dice coefficients for both normal and abnormal cases, making it the most effective model.

**Figure 5:**
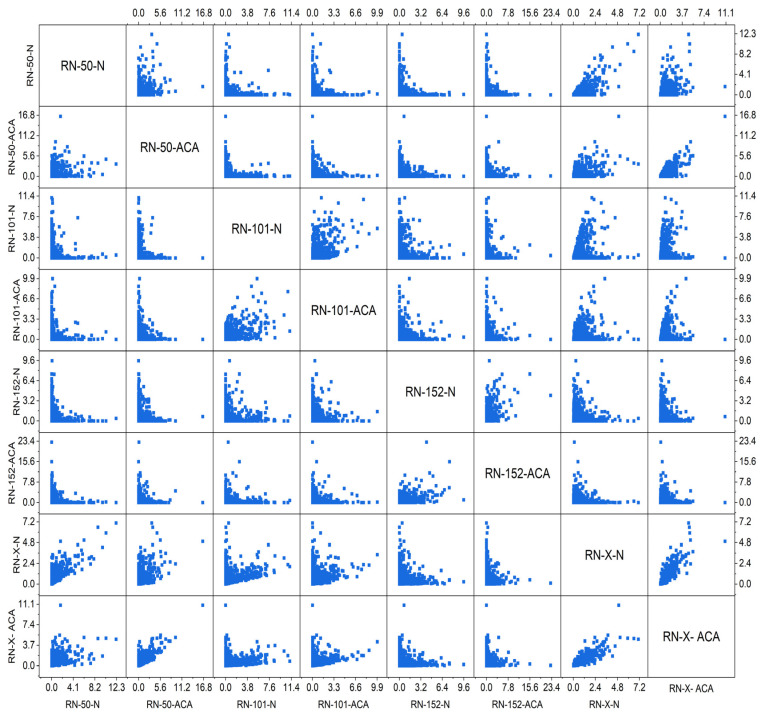
Scatterplot matrix of ResNet-50, ResNet-101, ResNet-152, and ResNet-X for Cancerous and Non-Cancerous Data.

Figure 5 is the scatter plot matrix, which provides insights into feature relationships across different models, including ResNet-50, ResNet-101, ResNet-152, and the proposed RN-X method. ResNet-50 (RN-50-N and RN-50-ACA) exhibits a relatively compact feature distribution, indicating limited complexity in extracted features. ResNet-101 (RN-101-N and RN-101-ACA) shows a broader spread, capturing more diverse patterns compared to RN-50. ResNet-152 (RN-152-N and RN-152-ACA) further expands the feature space, suggesting that deeper network layers extract more complex and discriminative information. However, the proposed RN-X method (RN-X-N and RN-X-ACA) demonstrates a distinct feature distribution, influenced by deep-weighted averaging-based feature fusion (DWAFF). The ACA-based versions of all models (RN-50-ACA, RN-101-ACA, RN-152-ACA, and RN-X-ACA) introduce additional refinement, enhancing feature discrimination. Notably, RN-X-ACA exhibits the most unique distribution, reinforcing the effectiveness of feature fusion and optimization techniques in improving classification performance compared to standalone ResNet architectures.

**Figure 6:**
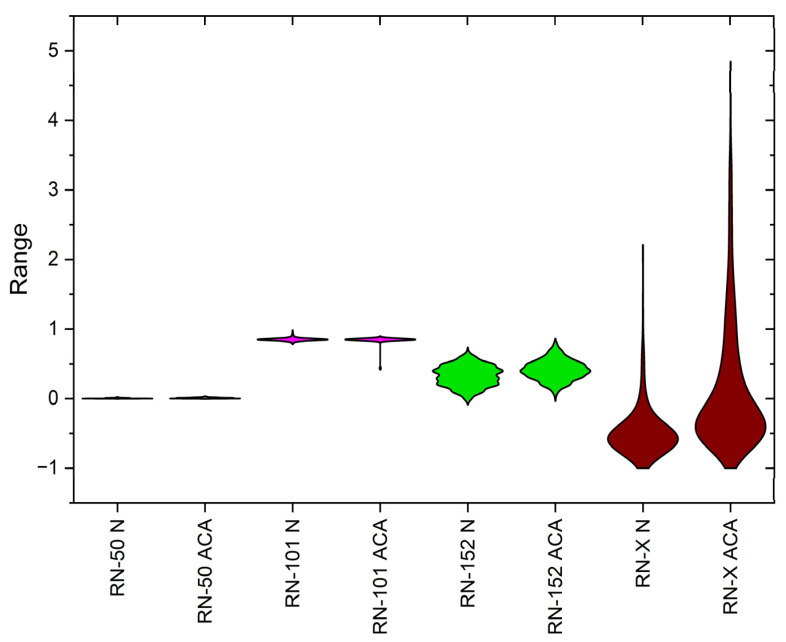
Violin plot of class distributions from deep features extracted via ResNet variants and DWAFF-RN-X fused features.

The violin plot in Figure 6 provides a detailed visualization of the feature distributions extracted by different ResNet models—ResNet-50, ResNet-101, ResNet-152, and the proposed ResNet-X—across normal (N) and abnormal (ACA) classes. ResNet-50 exhibits minimal variation with significant overlap between classes, indicating poor feature separability. ResNet-101 shows increased variation but still struggles with class differentiation due to substantial overlap. ResNet-152 presents more distinct distributions with reduced overlap, demonstrating improved feature extraction capabilities. In contrast, ResNet-X displays the widest distributions and the most pronounced class separation, suggesting superior feature discrimination. The progressive improvement from ResNet-50 to ResNet-X highlights the effectiveness of deeper architectures and feature fusion in enhancing classification performance for lung cancer diagnosis.

### 4.2. Selective Feature Pooling Layer

The selective feature pooling layer is designed to condense the features of histopathological images into compact feature vectors, enhancing classifier performance and promoting high generalization capability. In lung cancer diagnosis [29], these techniques enhance accuracy using bio-inspired optimization algorithms like PSO and RDO.

#### 4.2.1. Particle Swarm Optimization (PSO)

Particle swarm optimization (PSO), first proposed by Kennedy and Eberhart in 1995, mimics bird flock behavior to optimize problems. It begins by initializing particles and defining essential parameters [30]. The Algorithm 2 for the PSO as a Feature Selection with particle position and velocity updates is given below.
**Algorithm 2. Particle Swarm Optimization (PSO) as a Feature Selection****Step****Description****Details**Step 01Initialization    - Maximum iteration count: $k_{max}$    - Inertia weight range: ($w_{min}$, $w_{max}$)    - Acceleration coefficients: c1, c2    - Set the position of each particle randomly:$p_{i}^{k}=\left(p_{i1}^{k},p_{i2}^{k},...,p_{ix}^{k}\right)$ (14)    - Set the velocity of each particle randomly: $q_{i}^{k}=\left(q_{i1}^{k},q_{i2}^{k},...,q_{iy}^{k}\right)$ (15)    - Initialize the best position for an individual particle as $pbest_{i}=p_{i}^{k}$ and gbest = the best of all $pbest_{i}$.Step 02Iteration Loopfor k = 0 to k_max_ − 1 do:    for i = 1 to n do:       Calculate the inertia weight: $w_{i}=\frac{w_{max}-w_{min}}{k_{max}}\times k$ (16)       Update the velocity: $q_{i}^{k+1}=w_{i}q_{i}^{k}+c_{1}r_{1}\left(pbest_{i}-p_{i}^{k}\right)+c_{2}r_{2}\left(gbest_{i}-p_{i}^{k}\right)$ (17)       Update the position: $p_{i}^{k+1}=p_{i}^{k}+q_{i}^{k+1}$ (18)       Update $pbest_{i}$ if the new position surpasses the previous $pbest_{i}$       Update gbest if the new $pbest_{i}$ surpasses the current gbest.Step 03Output    Output the final gbest as the optimal solution.

In this study, the following parameter values are selected through an iterative process of experimentation and refinement: Inertia weight (wi)—between 0.45 and 0.9, maximum number of iterations—between 100 and 1000, random values (r1 and r2)—set to 0.85, cognitive component (c1) and social component (c2)—between 1.0 and 2.0.

#### 4.2.2. Red Deer Optimization (RDO)

Red Deer Optimization (RDA), introduced in 2016 [31], emulates the courtship rituals of Scottish red deer. The algorithm starts with an initial population of “red deer” (RDs). The best RDs, called “RD males”, are split into “commanders” and “stags”, based on their initial performance. Commanders and stags compete for harems, with successful stags potentially becoming commanders. Commanders pair with hinds in their harems and others, while stags mate with nearby hinds. This process blends exploration and exploitation, generating new solutions and allowing weaker solutions to evolve. In terms of dimensionality reduction, RDA uses this evolutionary process to refine and optimize the solution space by iteratively improving and filtering candidate solutions. The Algorithm 3 for the Red Deer Optimization (RDO) as a Feature Selection is as follows.
**Algorithm 3. Red Deer Optimization (RDO) as a Feature Selection****Step****Description****Details**Step 01Initial Population    - Define the solution space with dimensions:$Value=f\left(RedDeer\right)=f\left(S_{1},S_{2},S_{3},....,S_{N_{var}}()\right)$ (19)          Here, $S_{N_{var}}$ represents the array size, set to 50. Each component $S_{i}$ corresponds to a vector of values for each of the 50 images, as defined by the equation below:$\;S_{i}=\left[\theta_{1},\theta_{2},\theta_{3},\ldots ,\theta_{50}\right]=fori=1,2,3,....,S_{N_{var}}$
    - Initialize a random population of red deer (RDs).Step 02Roaring Stage    - For each male RD:    -- Calculate the new position based on fitness function (FF) value using$Male_{new}=\left\{\begin{array}{c} Male_{old}+a_{1}\times \left(\left(\left(UL-LL\right)*a_{2}\right)+LL\right),ifa_{3}\geq 0.5\\ Male_{old}-a_{1}\times \left(\left(\left(UL-LL\right)*a_{2}\right)+LL\right),ifa_{3}<0.5 \end{array}\right\}$ (20)Here, UL and LL represent the maximum and minimum boundaries of the search region, respectively. The factors a1, a2 and a3 are randomly selected from a uniform distribution between zero and one.    -- Update the RD position and evaluate its fitness.    -- Promote successful RDs to commander status if they show improved fitness.Step 03Competition Stage    - Each commander competes with random stags:    -- Compute new positions:$New1=\frac{\left(Com+Stag\right)}{2}+b_{1}\times \left(\left(\left(UL-LL\right)*b_{2}\right)+LL\right)$ (21)$New2=\frac{\left(Com+Stag\right)}{2}-b_{1}\times \left(\left(\left(UL-LL\right)*b_{2}\right)+LL\right)$ (22)Here $b_{1}$ and $b_{2}$ are the random numbers between 0 and 1 by uniform distribution function.    -- Select the position with the best fitness function (FF) to update the commander status.Step 04Harem Creation Phase    - For harems with    -- A commander and several hinds based on the commander’s fitness.    -- Calculate the number of hinds as:$N.harem_{n}=round\left\{P_{n}\cdot N_{hind}\right\}$ (23)    -- Stags do not participate in harems.Step 05Mating Phase    - Commander Mating Within Harems: Each commander mates with a proportion (α) of its hinds    - Commander Expansion Beyond Harems: Commanders mate with a percentage (β) of hinds from other harems. The parameter (β) ranges from 0 to 1.    - Stag Mating: Stags mate with the closest hind.Step 06Offspring Creation    - Generate new offspring using:$offspring=\frac{\left(Com+Hind\right)}{2}+\left(UL-LL\right)*c$ (24)where offspring is the new offspring RD, c is randomly chosen between 0 and 1. For -Stage mating, replace Com with Stag.Step 07Next-Generation Solution    - Retain a percentage of the best male RDs.    - Select hinds and offspring for the next generation using fitness-based methods.Step 08Stopping Criterion    - RDO’s stopping criteria include the following: 1. Fixed number of iterations. 2. Achievement of a quality threshold. 3. Exceeding a time limit.

The parameters of the RDO algorithm are described in the following table, Table 3.

#### 4.2.3. Entropy-Based on Statistical Analysis

In biomedical applications, entropy has emerged as a widely used approach. When applied to feature selection, entropy-based techniques assess the relevance and significance of selected features by quantifying the amount of information each feature contributes to predicting the target variable. In this study, the selected features from the normal and abnormal classes are evaluated using Shannon Entropy and Fuzzy Entropy.

##### Approximate Entropy

Approximate Entropy is a statistical method for measuring the regularity and unpredictability of variations in time-series data [32]. It calculates the difference between the natural logarithms of repeating patterns of length n and n + 1 using the following formula:

(25) $$Approximate\;Entropy\;(AE)=\ln\left(\frac{b_{n}(r)}{b_{n+1}(r)}\right)$$

Here, n is the input feature length, and $b_{n}(r)$ is the mean of all $b_{in}(r)$ ranges. $b_{in}(r)$ is given as

(26) $$b_{in}(r)=\frac{m_{in}(r)}{M-n+1}$$

In the input vector $V_{m}$ of length $\left[V_{m}(1),V_{m}(2),...,V_{m}(M-n+1)\right]$, $b_{in}(r)$ represents the number of features. A higher approximate entropy value indicates that the input feature vectors are more complex and less predictable.

##### Shannon Entropy

The Shannon Entropy of a random variable, S, containing values $s_{1},s_{2},...,s_{m}$ is determined by

(27) $$Shannon\;Entropy\;(SE)=-\sum_{n=1}^{m}p(s_{m})\cdot \log p(s_{m})$$

Here, $p(s_{m})$ represents the $s_{m}$ probability function. If the entropy score is high, it means that the outcome is hard to predict because it is uncertain.

##### Fuzzy Entropy

Fuzzy Entropy, a statistical method used to quantify the uniformity of input feature vectors [33], is defined by the following formula:

(28) $$Fuzzy\;Entropy\;(FE)=\ln\left(\frac{\phi^{n}}{\phi^{n+1}}\right)$$

Here, $\phi^{n}=\frac{1}{M-n}\sum_{p=1}^{M-n}\sum_{q=1,q\neq p}^{M-n}\frac{F_{pq}^{n}}{M-n-1}$, and $F_{pq}^{n}$ represents the membership value of the fuzzy set, and M is the total number of data points.

Table 4 compares the feature selection methods PSO and RDO in terms of entropy-based statistical parameters such as approximate entropy, Shannon entropy, and fuzzy entropy. Approximate entropy measures the regularity and predictability of time-series data. PSO, with lower approximate entropy values (1.2385 for N and 1.7816 for ACA) compared to RDO (2.0123 for N and 2.4893 for ACA), indicates more regularity and less complexity. RDO, with higher values, suggests greater variability and less predictability, possibly capturing more nuanced features. Shannon entropy reflects uncertainty or information content. RDO’s higher values (5.0821 for N and 5.8982 for ACA) show greater complexity and feature diversity compared to PSO’s lower values (3.8523 for N and 4.9891 for ACA), which suggest more structured data but less feature variety. Fuzzy entropy, which measures complexity in a fuzzy system, is higher for RDO (0.7283 for N and 0.9182 for ACA), indicating more ambiguity in feature relationships. PSO’s lower values (0.4862 for N and 0.5231 for ACA) suggest clearer, better-defined relationships with less uncertainty.

Table 5 compares the choice of hyperparameters of ResNet-50, ResNet-101, ResNet-152, and the proposed DWAFF method. All models use the Adam optimizer with increasing momentum values from 0.8 in ResNet-50 to 0.95 in DWAFF. The initial learning rate decreases progressively from 0.05 in ResNet-50 to 0.001 in DWAFF, with each model employing a different learning rate decay schedule. The learning rate is reduced by a factor of 10 at specified epochs, with ResNet-50 decaying every 4 epochs, ResNet-101 every 6, ResNet-152 every 8, and DWAFF every 10. Weight decay also decreases from 0.0005 in ResNet-50 to 0.00005 in DWAFF. All models maintain a batch size of 128. While the ResNet models utilize global average pooling, DWAFF incorporates a novel feature fusion approach. Each model is trained for 16 epochs, with a progressively decreasing learning rate schedule, where DWAFF exhibits a more gradual decay compared to the ResNet variants.

### 4.3. Classification Layer

Classifiers are essential for categorizing data, aiming for high accuracy and minimal errors while balancing computational complexity. This study utilized the following classifiers in the classification layer part of the ResNet architectures.

#### 4.3.1. Support Vector Machine (SVM)

SVM is a set of supervised learning methods utilized for categorization, prediction, and anomaly detection due to its scalability and high performance [34]. Linear SVMs use a maximum-margin hyperplane (either hard or soft margin), while non-linear SVMs apply kernel functions for classification. The hyperplane is determined by

(29) $$Minimize,\frac{1}{2}{\left\|w\right\|}^{2}+M\sum_{j=1}^{n}\mu_{j}$$

It is subject to $z_{j}{\left(w^{N}x_{j}+f\right)}^{3}\geq 1-\mu_{j},\mu_{j}\geq 0.$ Here, w is the vector that is perpendicular to the hyperplane, $x_{j}$ is a data point, f∈R is a scalar, and $\mu_{j}$ are slack variables penalizing misclassifications. The decision function is $w^{N}x_{j}+f$. Various kernels such as linear, polynomial, RBF, and sigmoid are used in SVMs. This study uses the SVM-RBF kernel to enhance classification accuracy.

#### 4.3.2. Decision Tree (DT)

A decision tree (DT) is a flexible algorithm for categorization and regression, using a tree structure with decision nodes based on features and leaf nodes for outcomes [34]. Starting at the root, the tree is traversed to make predictions. Nodes split data by feature and threshold, while leaf nodes provide final predictions. Key metrics for node impurity include the following:

(30) $$Information\;Gain\;\left(I,F\right)=Entropy\;(I)-\sum_{v\in values(F)}\frac{\left|I_{v}\right|}{I}*Entropy$$

where F represents the feature, I denotes the collection of instances at the node, and $I_{v}$ is the subset of instances with feature F has the value v. Gini impurity is given as follows:

(31) $$Gini\;(p)=1-\sum_{k=1}^{C}(p_{k}{)}^{2}$$

where $p_{k}$ is the frequency of class k, and C is the number of classes. The objective is to find the feature and threshold that minimize impurity, with the optimal split S given by the following:

(32) $$S\left(I\right)={argmax}_{x,t}\;impurity\;\left(I\right)-\sum_{v\in values\left(F\right)}\frac{\left|I_{v}\right|}{I}*impurity\;(I_{v})$$

Here, the values of x and t maximize the above $S\left(I\right)$ expression, impurity (I) and $impurity\;\left(I_{v}\right)$ denotes the impurity of the current node and subset $I_{v}$, $\frac{\left|I_{v}\right|}{I}$ denoted the proportion of samples in subset relative to the total samples.

#### 4.3.3. Random Forest (RF)

The random forest algorithm excels in image classification due to its accuracy and robustness [35]. It uses multiple independent decision trees, with key parameters including the number of trees and features considered by each tree. The final prediction is made by combining the decision from all trees with the following formula:

(33) $$f={\mathrm{argmax}}_{k}\sum_{j=1}^{D}p_{j}=l$$

where f is the final prediction, D is the number of trees, $p_{j}$ is the prediction from the $j^{th}$ tree, and l is the class label.

#### 4.3.4. K-Nearest Neighbor (KNN)

The KNN algorithm determines the category of a data point by comparing its distance to the K closest points in the training data and assigns it to the class that appears most frequently among these neighboring points. It requires no separate training phase and uses the entire dataset for classification. In weighted KNN, neighbors are weighted inversely to their distance from the query point [36]. The distance between two points $Z_{1}=(z_{11},z_{12},...,z_{1n})$ and $Z_{2}=(z_{21},z_{22},...,z_{2n})$ is calculated as follows:

(34) $$dist(Z_{1},Z_{2})=\sqrt{\sum_{i=1}^{n}(z_{1i}-z_{2i}{)}^{2}}$$

In weighted KNN, $w_{i}$ is given by $w_{i}=\frac{1}{d\left(z_{1}-z_{2}\right)+\in }$, with a small constant ∈ added to avoid division by zero. The classification of a query point is determined by the most common class among its K closest neighbors,

(35) $$\hat{b}={\mathrm{argmax}}_{c}\sum_{i\in S_{K\left(z\right)}}w_{i}\cdot I\left(b_{i}=c\right)$$

Here $S_{K\left(z\right)}$ represents the set of K nearest neighbors, $b_{i}$ is the class label of neighbor $b_{i}$, and $I\left(\cdot \right)$ is the indicator function. This study uses k = 5 with mixed Euclidean distance to improve classification accuracy by weighing closer neighbors more heavily.

#### 4.3.5. Softmax Discriminant Classifier (SDC)

The SDC identifies and verifies the class of a test sample [37] by measuring its distance to training samples within each class. Given a training set, $S=[S_{1},S_{2},....,S_{q}]\in R^{a\times b}$, where $S_{q}=[S_{1}^{q},S_{2}^{q},.....,S_{b_{q}}^{q}]\in R^{a\times b_{q}}$ contains $b_{q}$ samples from class q, with $\sum_{j=1}^{k}m_{j}=m$. The samples used for testing are presumed to be $S\in R^{a\times 1}$, and then SDC is defined as

(36) $$h(y)=\arg\underset{j}{max}S_{w}^{j}$$

(37) $$h(y)=\arg\underset{j}{max}\log\left(\sum_{n=1}^{b_{i}}\exp\left(-\lambda ||v-v_{n}^{j}|{|}_{2}\right)\right)$$

where $S_{w}^{j}$ measures the distinction between the test sample and class *j*. A penalty cost, λ > 0, is applied. If *v* and *v_n_* are similar, and y belongs to class *i*, $||v-v_{n}^{j}|{|}_{2}$ is close to zero, making $S_{w}^{j}$ approach its maximum value.

#### 4.3.6. Multi-Layer Perceptron (MLP)

Multilayer perceptron’s (MLPs) are used for function approximation tasks like regression [38]. The MLP structure consists of an input layer with n nodes, an intermediate layer, and an output layer. Input–output pairs are denoted as $\left(a_{m},b_{m}\right),\;for\;m=1,2,...,p$, where $a_{m}=(a_{m1},a_{m2},....,a_{mq})$ is the input vector, and $b_{m}$ is the target output; the output $z_{mp}$ of the k-th hidden node is computed as follows:

(38) $$z_{mp}=f_{s}\left(\sum_{j=1}^{n}w_{jk}a_{mj}+\theta_{k}\right)$$

The final output $z_{m}$is given by

(39) $$z_{m}=f_{s}\left(\sum_{p=1}^{j}w_{p}z_{mp}+\theta \right)$$

where j represents the number of hidden units, *θ* denotes the bias at the output layer, and $w_{p}$ represents the weight connecting the k-th hidden unit to the output layer. This configuration results in (*n* + 2) j + 1 connections. The cost function for training the MLP is

(40) $$F=\frac{1}{2}\sum_{k=1}^{z}{\left(q_{m}-z_{m}\right)}^{2}$$

In this study, a three-layer architecture was utilized, recognized for its effectiveness in approximating continuous functions [39].

#### 4.3.7. BLDC

The Bayesian Linear Discriminant Classifier (BLDC) employs the Fisher linear discriminant alongside Bayes decision rule to reduce the probability of classification errors [40], effectively regularizing high-dimensional signals and enhancing computational efficiency. In Bayesian regression, the target a is defined as follows:

(41) $$a=q^{T}s+n$$

where *q* is the weight vector, and *n* is white Gaussian noise. The weighted likelihood function is

(42) $$p\left(\frac{D}{\beta ,q}\right)={\left(\frac{\beta }{2\pi }\right)}^{\frac{M}{2}}\exp\left(\frac{-\beta }{2}{\left\|V^{C}q-a\right\|}^{2}\right)$$

Here, a is the target value, V is the matrix of training feature vectors, and D combines V and a. β is the inverse noise variance, and C is the number of training samples. The prior distribution is given by

(43) $$p(q|\alpha )={\left(\frac{\alpha }{2\pi }\right)}^{\frac{N}{2}}{\left(\frac{\varepsilon }{2\pi }\right)}^{\frac{1}{2}}\exp\left(\frac{-q^{C}R^{\prime }(\alpha )q}{2}\right)$$

where N is the feature size, and $R^{\prime }(\alpha )$ denotes the (N+1) dimensional regularization diagonal matrix, represented as follows:

(44) $$R^{\prime }(\alpha )=\left[\begin{array}{ccc} \alpha & \cdots & 0\\ \vdots & \ddots & \vdots \\ 0 & \cdots & \varepsilon \end{array}\right]$$

The posterior distribution of s is

(45) $$p\left(\frac{q}{\beta ,\alpha ,D}\right)=\frac{p\left(\frac{D}{\beta ,s}\right)p\left(\frac{s}{\alpha }\right)}{\int p\left(\frac{D}{\beta ,s}\right)p\left(\frac{s}{\alpha }\right)}$$

This posterior distribution is Gaussian with covariance matrix *H* and mean vector U:

(46) $$U=\beta {\left(\beta VV^{C}+R^{\prime }(\alpha )\right)}^{-1}Va$$

(47) $$H={\left(\beta VV^{C}+R^{\prime }(\alpha )\right)}^{-1}$$

For the predictive variation of $\hat{q}$, the distribution on the regression target is

(48) $$p\left(\frac{\hat{a}}{\beta ,\alpha ,\hat{q},D}\right)=\int p\left(\frac{\hat{a}}{\beta ,\alpha ,\hat{q},s}\right)p\left(\frac{s}{\beta ,\alpha ,D}\right)ds$$

This predictive distribution is also Gaussian with mean and variance, as follows:

(49) $$\mu =v^{C}\hat{q}$$

(50) $$\delta^{2}=\left(\frac{1}{\beta }\right)+{\hat{q}}^{C}H\hat{q}$$

## 5. Results and Discussion

This study used deep learning-based feature extraction and feature fusion techniques, along with feature selection using PSO and RDO, to categorize histopathological images of lung cancer on a Windows 11 workstation with an AMD Ryzen 7 5700 G processor and integrated Radeon Graphics of 1 TB, running MATLAB 2018a.

### 5.1. Training and Testing of the Classifiers

In this study, K-fold cross-validation was used for classification. For instance, with K = 10, the dataset was divided into 10 equal segments, where each segment was used once as the test set and the remaining nine as the training set. Performance metrics were averaged across iterations. Different K values, such as 2, 4, 5, 8, and 10, were evaluated. The training data were partitioned into smaller batches, and classifiers such as SVM, DT, RF, KNN, and BLDC were trained iteratively on these smaller batches, while MLP and SDC were trained directly over epochs. After each epoch, performance was evaluated on both training and validation sets, and accuracy was recorded. Finally, the training and validation accuracies were plotted to visualize performance over epochs. Training stopped after a maximum of 15 epochs or when accuracy levels suggested potential overfitting. Testing ended once all batches were processed. Higher accuracy and lower error rates indicate better classifier performance. Table 6 lists the parameters selected for various classifiers, chosen through trial and error, with a maximum of 15 epochs to prevent overfitting.

### 5.2. Standard Benchmark Metrics of the Classifiers

In this study, several transfer learning models were assessed using a confusion matrix. The evaluation process involved using 90% of the input features for training and setting aside 10% for testing. In the context of lung cancer detection, the clinical scenarios related to the confusion matrix were defined as follows: true positive (TP), correctly identifying a patient’s tumor as benign. True negative (TN): correctly identifying a patient as having adenocarcinoma. False positive (FP): incorrectly classifying a patient’s adenocarcinoma as benign. False negative (FN): misclassifying a patient’s benign tumor as adenocarcinoma. The performance of the classifiers is evaluated using metrics such as accuracy, error rate, F1 score, Matthews correlation coefficient (MCC), Jaccard index, G-mean, and kappa. The mathematical formulations for these metrics are detailed in Table 7.

### 5.3. Performance Analysis of the Classifiers in Terms of Accuracy for Different K Values

In this study, the performance of seven classifiers—SVM, KNN, random forest, decision tree, softmax discriminant, MLP, and BLDC—was evaluated for cancer image classification across K values of 2, 4, 5, 8, and 10.

**Figure 7:**
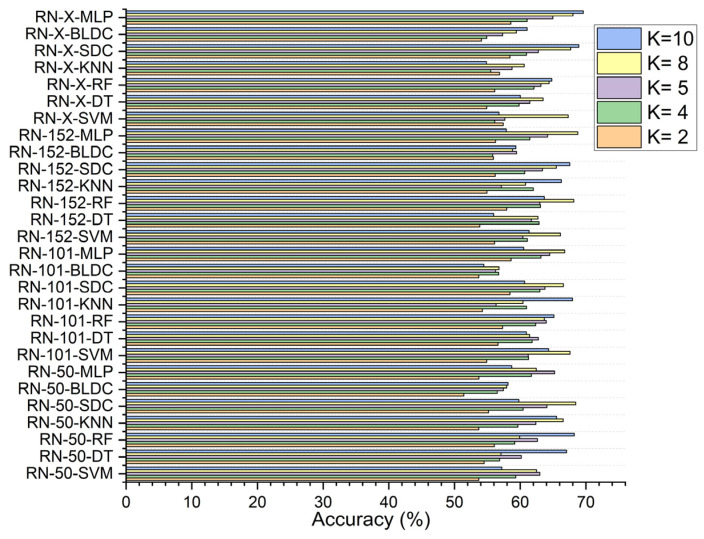
Classifier performance in accuracy for K = 2, 4, 5, 8, 10—without segmentation.

In the first scenario, without segmentation, as shown in Figure 7, the ResNet-X based feature fusion technique, combined with an MLP model, achieved an accuracy of 58.610% at K = 2 and 63.150% at K = 4. The ResNet-50-based feature extraction with the MLP model reached its highest accuracy of 65.230% at K = 5, while the ResNet-152-based feature extraction with the MLP model attained a peak accuracy of 68.783% at K = 8. Additionally, the ResNet-X based feature fusion technique combined with the MLP model achieved a top accuracy of 69.610% at K = 10.

**Figure 8:**
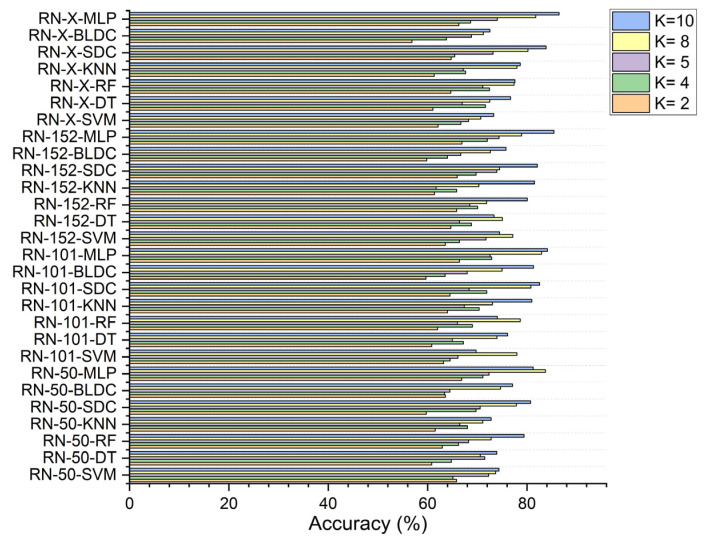
Classifier performance in accuracy for K = 2, 4, 5, 8, 10—with segmentation.

In the second scenario, with segmentation as shown in Figure 8, ResNet-152-based feature extraction combined with the MLP model achieved 66.920% accuracy at K = 2 and 74.380% at K = 5. The ResNet-101 based feature extraction with the MLP reached 72.910% at K = 4, while the ResNet-50 based feature extraction with the MLP peaked at 83.730% at K = 8. Additionally, the ResNet-X based feature fusion technique combined with the MLP attained a top accuracy of 86.460% at K = 10.

**Figure 9:**
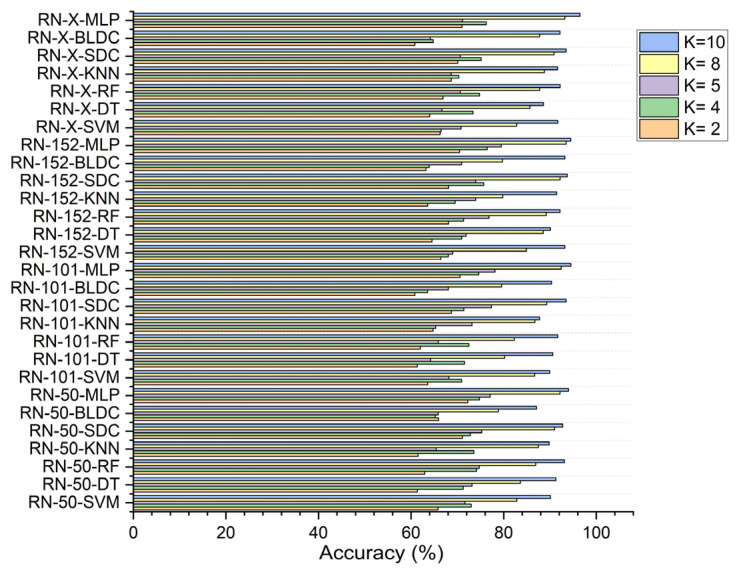
Classifier performance in accuracy for K = 2, 4, 5, 8, 10—with segmentation and PSO feature selection.

In the third scenario, with segmentation and feature selection, applying PSO for feature selection resulted in a ResNet-50-based feature extraction with the MLP achieving 72.250% accuracy at K = 2. The ResNet-152-based feature extraction with the MLP attained 76.432% at K = 4, 79.490% at K = 5, and 93.508% at K = 8, while the ResNet-X-based feature fusion technique combined with the MLP reached 96.490% at K = 10, as shown in Figure 9.

**Figure 10:**
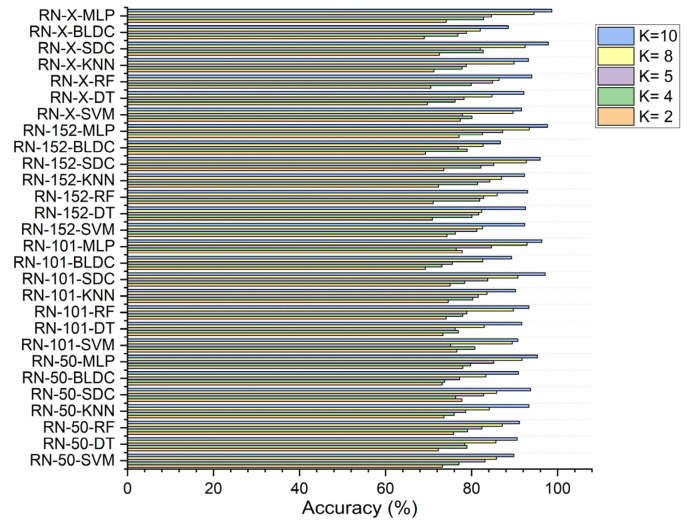
Classifier performance in accuracy for K = 2, 4, 5, 8, 10—with segmentation and RDO feature selection.

Using RDO for feature selection, the ResNet-50 based feature extraction with the MLP achieved 77.980% accuracy at K = 2. The ResNet-152-based feature extraction with the MLP reached 87.240% at K = 5, while the ResNet-X based feature fusion technique combined with the MLP recorded 82.810% at K = 4, 94.531% at K = 8, and 98.680% at K = 10, as shown in Figure 10.

Across all scenarios, ResNet-X-based feature fusion technique combined with the MLP consistently achieved the highest accuracy, demonstrating its effective deep-weighted averaging feature fusion capabilities.

### 5.4. Performance Analysis of Classifiers for K = 10

**Figure 11:**
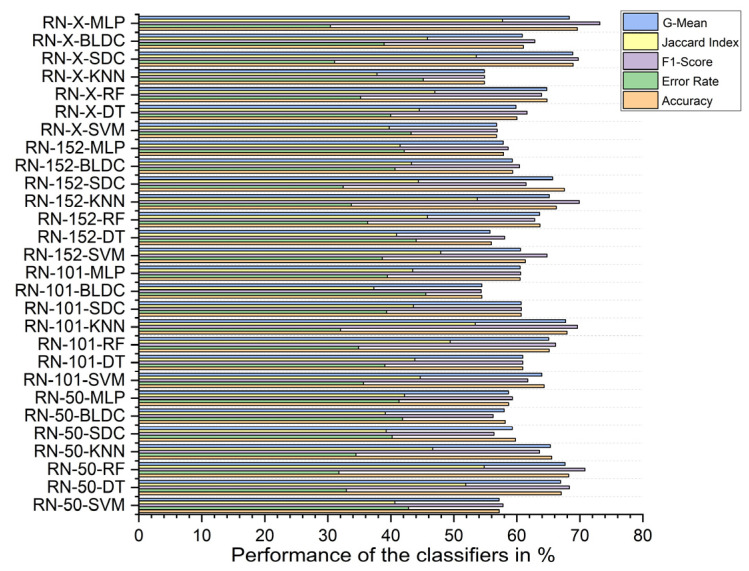
Classifier performance when K = 10—without segmentation.

Without segmentation, the ResNet-X-based feature fusion technique combined with MLP classifiers achieved the highest performance, with an accuracy of 69.610%, an F1 score of 73.184%, a Jaccard index of 57.709%, a G-Mean of 68.321%, and an error rate of 30.390%. In contrast, the ResNet-101-based feature extraction with BLDC classifiers had the lowest accuracy at 54.450%, an F1 score of 54.345%, a Jaccard index of 37.310%, a G-mean of 54.449%, and a high error rate of 45.550%, as shown in Figure 11.

**Figure 12:**
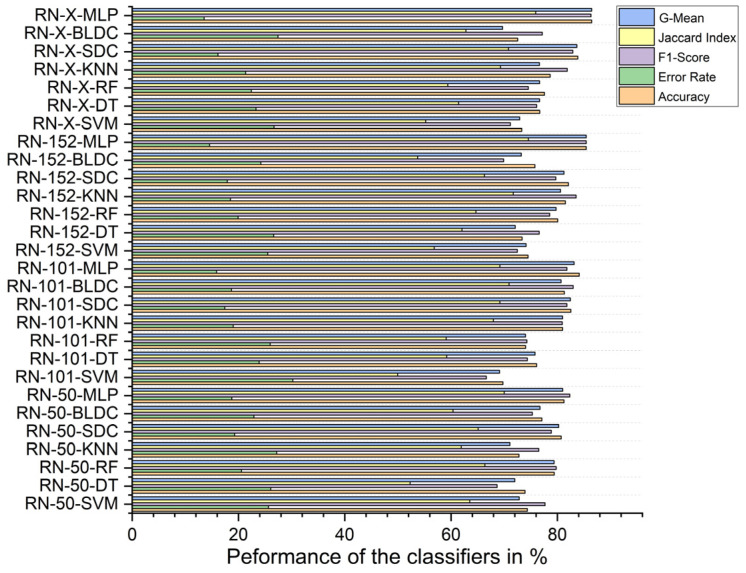
Classifier performance when K = 10—with segmentation.

With segmentation, as shown in Figure 12, the ResNet-X-based feature fusion technique with MLP classifiers performed best, achieving 86.460% accuracy, an F1 score of 86.317%, a Jaccard index of 75.928%, a G-mean of 86.453%, and an error rate of 13.540%. On the other hand, the ResNet-101-based feature extraction combined with SVM classifiers achieved the lowest performance, with 69.760% accuracy, an F1 score of 66.622%, a Jaccard index of 49.950%, a G-mean of 69.123%, and an error rate of 30.240%.

**Figure 13:**
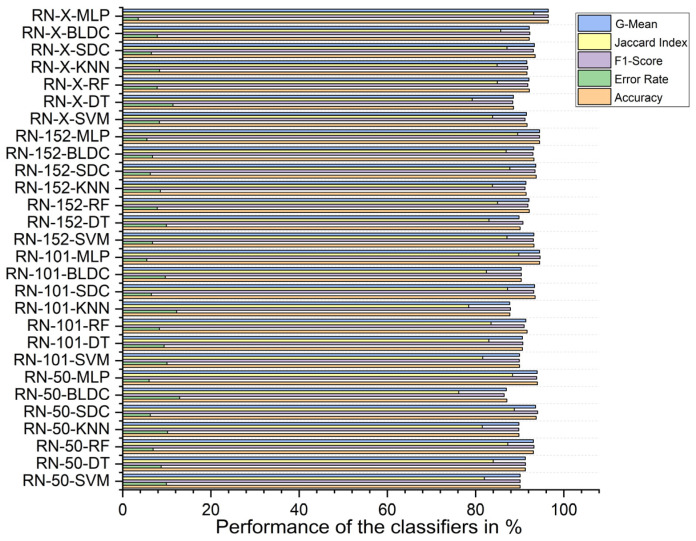
Classifier performance when K = 10—with segmentation and PSO feature selection.

Using PSO feature selection, the ResNet-X-based feature fusion technique combined with MLP classifiers achieved the highest accuracy at 96.490%, with an F1 score of 96.476%, a Jaccard index of 93.192%, a G-mean of 96.489%, and an error rate of 3.510%. In contrast, the ResNet-50-based feature extraction with BLDC classifiers achieved the lowest accuracy at 87.100%, an F1 score of 86.483%, a Jaccard index of 76.186%, a G-mean of 86.980%, and an error rate of 12.900%, as shown in Figure 13 below.

**Figure 14:**
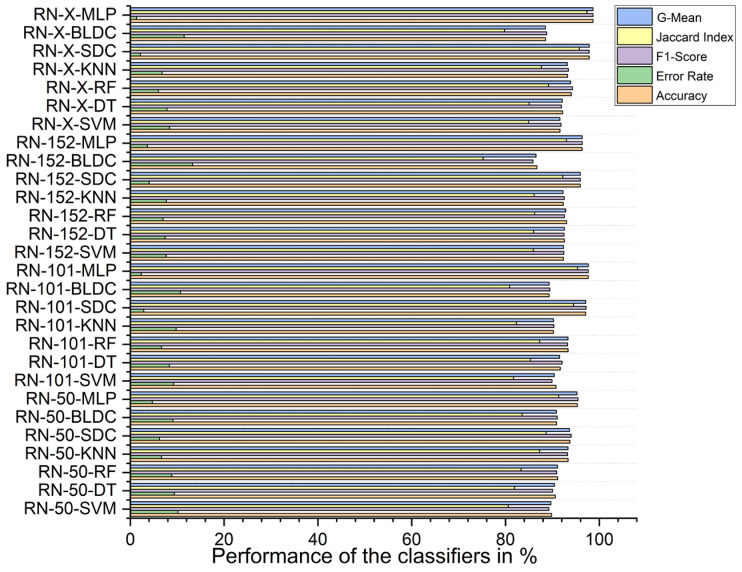
Classifier performance when K = 10—with segmentation and RDO feature selection.

Using RDO feature selection, the ResNet-X-based feature fusion technique with MLP classifiers again achieved the highest performance, with 98.680% accuracy, an F1 score of 98.669%, a Jaccard index of 97.347%, a G-mean of 98.677%, and an error rate of 1.320%. The lowest accuracy was recorded for the ResNet-152-based feature extraction with BLDC classifiers, which had an accuracy of 86.710%, an F1 score of 85.863%, a Jaccard index of 75.228%, a G-mean of 86.502%, and an error rate of 13.290%, as shown in Figure 14 below.

**Figure 15:**
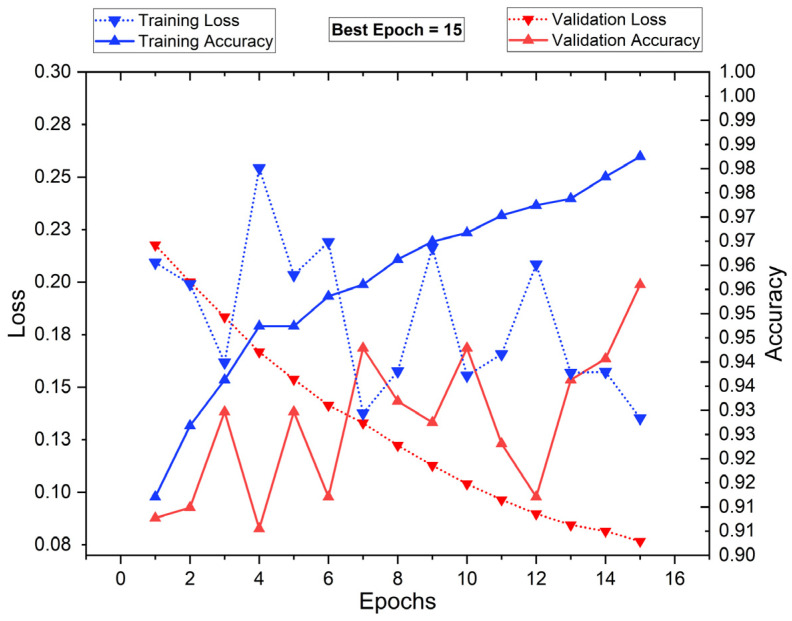
Training vs. validation performance plot: with segmentation and PSO FS.

Figure 15 presents the training and validation loss (dotted lines) alongside training and validation accuracy (solid lines) over 16 epochs. The blue markers represent training performance, while the red markers indicate validation performance. The training accuracy shows a consistent upward trend, reaching nearly 98.8% by epoch 16, while the training loss decreases, reflecting proper model learning. However, validation accuracy fluctuates, with its highest peak at epoch 15, suggesting optimal generalization at that point. The validation loss decreases initially but shows oscillations, indicating some variance in performance. The best epoch is identified as 15, balancing high accuracy and low loss before potential overfitting. This analysis highlights the importance of early stopping to ensure robust model generalization.

**Figure 16:**
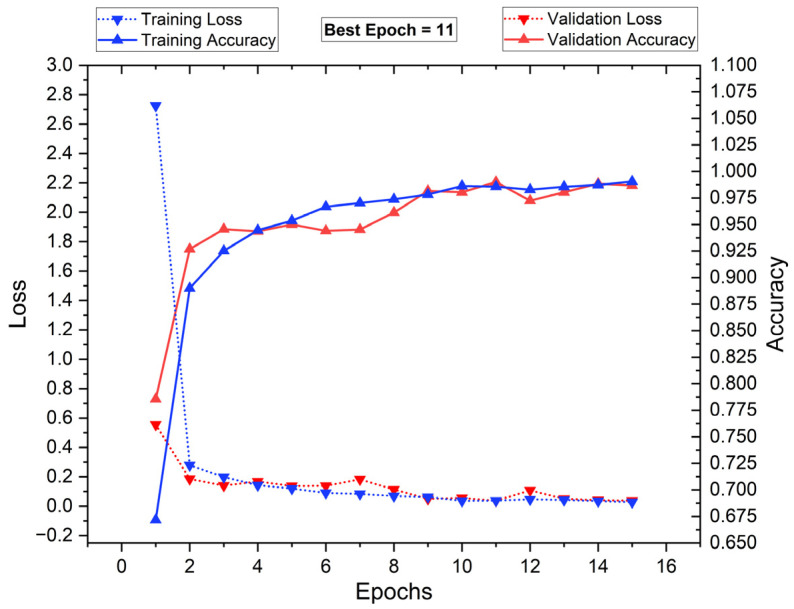
Training vs. validation performance plot: with segmentation and RDO FS.

Figure 16 illustrates the training and validation loss (dotted lines), along with training and validation accuracy (solid lines) over 16 epochs. The blue markers represent training metrics, while the red markers indicate validation performance. Initially, training loss is high but drops sharply within the first few epochs, stabilizing around epoch 4. The validation loss follows a similar pattern, indicating a smooth learning process. Training and validation accuracy increase rapidly, reaching nearly 100% by epoch 11, which is identified as the best epoch. Beyond this point, the accuracy plateaus, suggesting that further training does not yield significant improvements. The close alignment of training and validation metrics suggests minimal overfitting, demonstrating strong generalization.

**Figure 17:**
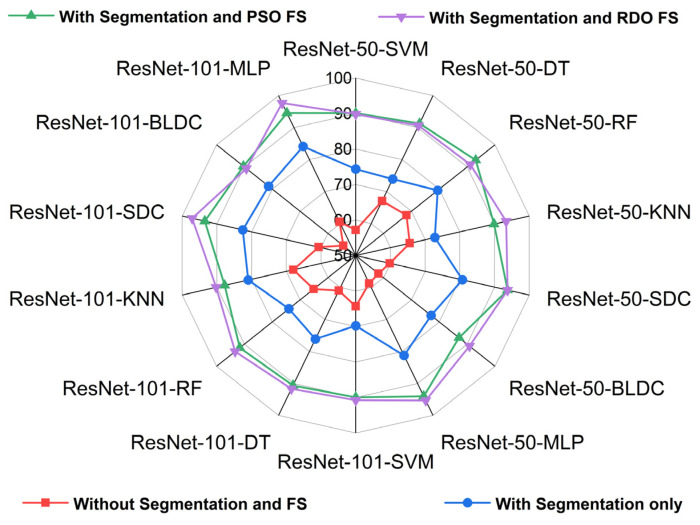
Radar plot for performance analysis of ResNet-50 and ResNet-101 with classifiers for K = 10.

**Figure 18:**
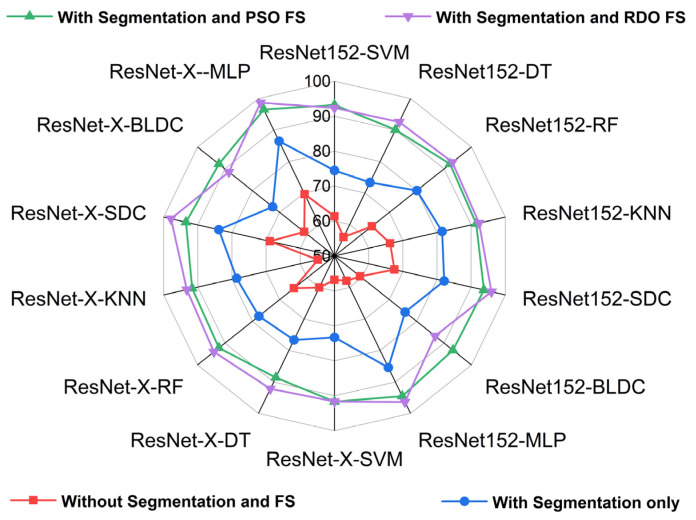
Radar plot for performance analysis of ResNet-152 and DWAFF-based ResNet-X with classifiers for K = 10.

Figure 17 and Figure 18 show radar plots that evaluate the performance of classifiers using ResNet-based deep feature extraction and optimization techniques in the selective feature pooling layer for feature selection, with K = 10 in K-fold cross-validation. The analysis compares input images with segmentation, without segmentation, and with segmentation combined with PSO- and RDO-based feature selection. The results indicate that ResNet-X-MLP achieves the highest accuracy of 69.610% without segmentation and 86.460% with segmentation alone. When feature selection is applied, ResNet-X-MLP achieves 96.490% with PSO and 98.680% with RDO. RDO shows more consistent performance across epochs, while PSO demonstrates instability, as shown in Figure 15 and Figure 16, making ResNet-X-MLP with RDO the more stable choice for classification.

**Figure 19:**
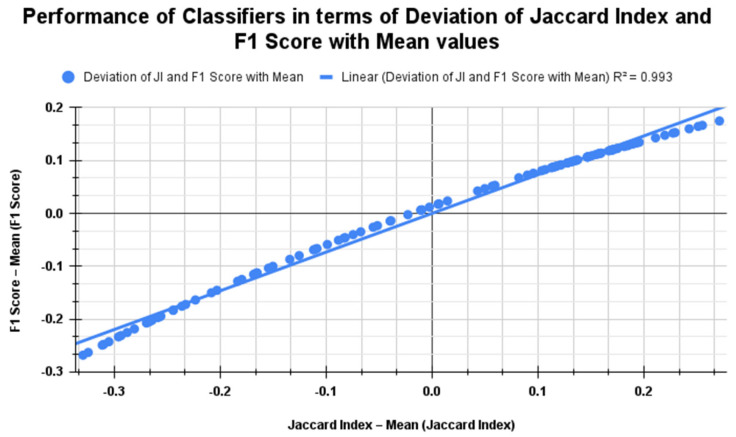
Comparison of classifier performance using Jaccard index vs. F1 score metrics for all three cases when K = 10.

Figure 19 shows the Jaccard index and F1 score performance for K = 10 across the classifiers. It reveals a strong positive linear relationship in scenarios with no segmentation, with segmentation alone, and with both segmentation and feature selection using PSO and RDO. The R^2^ value of 0.993 indicates an almost perfect linear correlation. The regression line $y=0.933\cdot x+1.72\times 10^{-16}$ shows that the F1 Score deviation increases slightly less than the Jaccard index deviation, with a near-zero intercept suggesting minimal deviation from the mean. Table 8 details the previous work carried out in lung cancer detection on various datasets.

### 5.5. Major Outcomes and Limitations

This research may have faced limitations due to the specific histopathological images used, which might not be generalizable to other image types or healthcare settings. Issues such as reliance on intensity values from segmented images, outliers, and data overlap could impact classification accuracy. Despite these challenges, the study’s approach, which combines various feature extraction methods, shows promise for identifying cancerous cells in histopathological images. A significant outcome is the creation of a comprehensive lung cancer screening database, which could enhance early detection and improve patient outcomes. Overall, this research provides valuable insights into early lung cancer detection and paves the way for further exploration.

### 5.6. Computational Complexity

This study evaluates the computational complexity of ResNet-50, ResNet-101, and ResNet-152-based feature extraction, as well as deep-weighted averaging-based feature fusion (DWAFF) features, in combination with feature selection techniques like PSO and RDO across various classifiers, using Big O notation.

In k-fold cross-validation, training on one-fold has a time complexity of $O\left(k\times T\right)$, as the model is trained k times. Complexity grows with input size n, where $O\left(1\right)$ signifies minimal complexity, while $O\left(\log n\right)$ denotes logarithmic growth. Table 9 details the computational complexity and execution times for pretrained transfer learning architectures with various classifiers and feature extraction methods. DWAFF-based ResNet-X fused features with an MLP classifier, using RDO feature selection, achieves the highest complexity at $O\;({4n}^{10}\log n)$ and the longest execution time of 480 s due to the extensive training across multiple layers.

## 6. Conclusions

Lung cancer represents a major worldwide health issue, leading significantly to illness and mortality. Although treatment advancements have been made, early detection and prevention remain vital for addressing this serious public health issue. This study implements the deep-weighted averaging-based feature fusion (DWAFF) technique on ResNet-50, ResNet-101, and ResNet-152 architectures for deep feature extraction. Additionally, a selective feature pooling layer is applied after feature extraction to reduce the feature set, which is then fed into seven classifiers for the effective classification of adenocarcinoma and benign images from the LC25000 dataset. Performance is measured using standard benchmark metrics, demonstrating strong results in classifying complex lung cancer images. Training and testing were conducted with K-fold cross-validation. The DWAFF-based ResNet-X fused features combined with MLP classifiers for the RDO feature selection method achieved the highest performance, with an accuracy of 98.68%, an F1 score of 98.67%, a Jaccard index of 97.37%, and a G-mean value of 98.68% at K = 10. Future research will focus on extending this approach to multiclass classification and other cancers, such as colon cancer, and exploring the incorporation of RNN models like LSTM and Bi-LSTM to further improve classification accuracy and support ongoing clinical monitoring.

## Figures and Tables

**Table 1:** Residual blocks of ResNet versions.

ResNet Architectures	A	B	C	D
RN-50	3	4	6	3
RN-101	3	4	23	3
RN-152	3	8	36	3

**Table 2:** Statistical parameters of extracted deep features and fused features of benign and malignant data.

Statistical Parameters	ResNet-50		ResNet-101		ResNet-152		DWAFF- ResNet-X	
	N	ACA	N	ACA	N	ACA	N	ACA
Mean	0.3384	0.3429	0.3444	0.3347	0.3508	0.3419	0.4538	0.4537
Variance	0.7147	0.8100	0.7920	0.8463	0.7987	0.8655	0.3807	0.4445
Skewness	5.4461	5.9408	5.4387	6.0005	5.5525	6.2248	3.7679	4.4868
Kurtosis	43.6833	52.9968	42.8377	52.9611	46.3895	60.2777	21.1486	33.4781
PCC	0.4994	0.5272	0.4958	0.5167	0.4944	0.5185	0.9386	0.9443
Dice Coefficient	0.7512	0.7043	0.8028	0.7557	0.8598	0.8011	0.9038	0.8572
CCA	0.7018		0.7532		0.8293		0.8816	

**Table 3:** Parameters of RDO.

S. No.	Parameters	Value	S. No.	Parameters	Value
1	Number of Populations	100	6	Beta	0.5
2	Simulation Time	13 (s)	7	Gamma	0.6
3	Number of Male RDs	12	8	Roar	0.23
4	Number of Hinds	58	9	Fight	0.47
5	Alpha	0.9	10	Mating	0.78

**Table 4:** Entropy-based statistical measures for PSO and RDO DR techniques.

Statistical Measures	PSO		RDO	
	N	ACA	N	ACA
Approximate Entropy	1.2385	1.7816	2.0123	2.4893
Shannon Entropy	3.8523	4.9891	5.0821	5.8982
Fuzzy Entropy	0.4862	0.5231	0.7283	0.9182

**Table 5:** Hyperparameters of ResNet Architectures with DWAFF Method.

Hyperparameters	ResNet-50	ResNet-101	ResNet-152	DWAFF (Proposed Method)
Optimizer	Adam	Adam	Adam	Adam
Momentum	0.8	0.85	0.9	0.95
Initial Learning Rate	0.05	0.03	0.01	0.001
Learning Rate Decay	1/10 every 4 Epochs	1/10 every 6 Epochs	1/10 every 8 Epochs	1/10 every 10 Epochs
Weight Decay	0.0005	0.0003	0.0001	0.00005
Batch Size	128	128	128	128
Pooling Type	Global Average Pooling	Global Average Pooling	Global Average Pooling	Feature Fusion (DWAFF)
Total Epochs	16	16	16	16
Learning Rate Schedule	0.05 → 0.005 (Epoch 4) → 0.0005 (Epoch 8) → 0.00005 (Epoch 12)	0.03 → 0.003 (Epoch 6) → 0.0003 (Epoch 12)	0.01 → 0.001 (Epoch 8) → 0.0001 (Epoch 16)	0.001 → 0.0005 (Epoch 10) → 0.0001 (Epoch 16)

**Table 6:** Selection of optimal parameters for the classifiers.

Classifiers	Description
SVM	Kernel function—RBF; support vector coefficient, α = 1.8; Gaussian function bandwidth (σ) = 98; bias term (b) = 0.012; convergence criterion—MSE.
KNN	K-5; distance metric—Euclidian; weight—0.52; criterion—MSE.
RF	Number of trees—150; maximum depth—15; bootstrap sample size—16; class weight—0.35.
DT	Maximum depth—14; impurity criterion—MSE; class weight—0.25.
SDC	λ—0.458, along with the average target values for each class being 0.15 and 0.85.
MLP	Learning rate—0.45; training method—LM; criterion—MSE.
BLDC	Mean µ and Covariance matrix H are calculated with a prior probability of 0.12; convergence criteria = MSE.

**Table 7:** Performance metrics of the classifiers with their significance.

Performance Metrics	Equation	Significance
**Accuracy (%)**	$Accuracy=\frac{TP+TN}{TP+TN+FP+FN}\times 100$	The overall accuracy of the classifier’s predictions.
**Error rate (%)**	$Err=\frac{FP+FN}{TP+TN+FP+FN}\times 100$	The ratio of misclassified instances
**F1 score (%)**	$F1=\frac{2TP}{2TP+FP+FN}\times 100$	The harmonic mean of precision and recall, reflecting the classification accuracy for a specific class
**MCC**	$MCC=\frac{TN\times TP-FN\times FP}{\sqrt{\left(TP+FP)(TP+FN)(TN+FP)(TN+FN\right)}}$	The Pearson correlation between the observed and predicted classifications
**Jaccard index (%)**	$Jaccard=\frac{TP}{TP+FP+FN}\times 100$	The proportion of predicted true positives to the sum of predicted true positives and actual positives, regardless of their true or predicted status
**G-mean (%)**	$g-mean=\sqrt{\left(\frac{TP}{TP+FN}\right)\times \left(\frac{TN}{TN+FP}\right)}\times 100$	A metric combines sensitivity and specificity into a singular value balancing both objectives
**Kappa**	$Kappa=\frac{\mathrm{Pr}\left(a\right)-Pr(e)}{1-Pr(e)}$	Evaluates how well the observed and predicted classifications align, reflecting the consistency of the classification outcomes

**Table 8:** Comparison of classifier performance with different datasets.

S. No.	Authors	Dataset Used	Classification Models	Accuracy (%)	Challenges
1	Jain et al. (2022) [41]	1500 images from LZ2500 dataset	Kernel PCA combined with faster deep belief networks	97.10%	Data availability, computational complexity and generalizability across different medical centers
2	Civit-Masot et al. (2022) [42]	15,000 images from LC25000 dataset	Custom architecture with three convolutional and two dense layers	99.69% with 50 epochs	Overfitting risk due to high accuracy, lack of clinical validation, dataset bias
3	Iftikhar Naseer et al. (2023) [43]	LUNA 16 Database	LungNet-SVM	97.64%	Limited dataset, potential model bias, difficulty in handling real-world noise in CT scans
4	Wang et al. (2023) [44]	993 WSIs from TCGA dataset	A novel multiplex detection-based MIL model	90.52%	Complexity in handling whole-slide images (WSIs), interpretability issues in MIL-based models
5	Mehedi Masud et al. (2021) [45]	LC25000 dataset	Custom CNN architecture consisting of three convolutional layers and one FC layer	96.33%	Lack of robustness to dataset variability, potential overfitting, limited feature extraction
6	Radical Rakhman Wahid et al. (2023) [46]	LC25000 Database	Customized CNN model	93.02%	Computational inefficiency, insufficient testing on real-world medical images
7	Gupta et al. (2022) [47]	TCGA dataset	Deep CNN	92%	High data variability in TCGA, lack of interpretability in deep CNNs
8	Liu et al. (2022) [48]	766 lung WSIs from First Hospital of Baiqiu’en and LC25000 dataset	SE-ResNet-50 with novel activation function CroRELU	98.33%	Computationally intensive, risk of overfitting with small dataset, limited clinical validation
9	Wang et al. (2023) [49]	988 samples with both CNV and histological data	LungDIG: combination of InceptionV3 with MLP	87.10%	Low accuracy compared to other models, difficulty in integrating CNV data with histological features
10	Mastouri et al. (2021) [50]	LUNA16 Database (3186 CT images)	BCNN [VGG16, VGG19]	91.99%	Pretrained VGG models may not generalize well, requires fine-tuning, dataset-specific performance
11	Phankokkruad (2021) [51]	LC25000 Database	Ensemble ResNet50V2	91% 90%	Ensemble models require higher computational resources, longer training time
12	Bukhari et al. (2020) [52]	CRAG Dataset	ResNet-50	93.91%	Requires large datasets to avoid overfitting, difficulty in domain adaptation
13	Sunila Anjum et al. (2023) [53]	LC25000 Database	EfficientNet (B0 to B7)	97%	EfficientNet may struggle with small datasets, needs proper tuning for histopathological images
14	Poonam Shourie et al. (2023) [54]	LC25000 Database	EfficientNet B7	98.49%	High computational cost, requires a large dataset for better generalization
15	Joge Diosdado et al. (2024) [55]	LungHist700	DNN and MIL	81–92%	Dataset size limitation, MIL-approach complexity, difficulty in model explainability
16	Karthikeyan Shanmugam, Harikumar Rajaguru This research	LC25000 Database	Feature extraction—RDO in selective feature layer—ResNet-X framework with MLP classifier	98.698%	—

**Table 9:** Computational complexity of the classifiers.

Deep Feature Extraction—Architectures	Classifiers	Without Segmentation	With Segmentation	With Segmentation and PSO Feature Selection	With Segmentation and RDO Feature Selection
ResNet-50	SVM	$O\;({2n}^{2}logn)$	$O\;({2n}^{3})$	$O\;({2n}^{5})$	$O\;({4n}^{5})$
	DT	O (log2n)	O (nlog2n)	$O\;(n^{3}log2n)$	$O\;(2n^{3}log2n)$
	RF	O (nlog2n)	$O\;(n^{2}log2n)$	$O\;(n^{4}log2n)$	$O\;(2n^{4}log2n)$
	KNN	$O\;(n^{2}logn)$	$O\;(n^{3}logn)$	$O\;(n^{5}logn)$	$O\;(2n^{5}logn)$
	SDC	$O\;(n^{3}logn)$	$O\;(n^{4}logn)$	$O\;(n^{6}logn)$	$O\;({2n}^{6}logn)$
	BLDC	$O\;(n^{2}logn)$	$O\;(n^{3}logn)$	$O\;(n^{5}logn)$	$O\;(2n^{5}logn)$
	MLP	$O\;(n^{5}logn)$	$O\;(n^{6}logn)$	$O\;(n^{8}logn)$	$O\;({2n}^{8}logn)$
ResNet-101	SVM	$O\;({2n}^{3}logn)$	$O\;({2n}^{4}logn)$	$O\;({2n}^{6}logn)$	$O\;({4n}^{6}logn)$
	DT	O (nlog2n)	$O\;(n^{2}log2n)$	$O\;(n^{4}log2n)$	$O\;(2n^{4}log2n)$
	RF	$O\;(n^{2}log2n)$	$O\;(n^{3}log2n)$	$O\;(n^{5}log2n)$	$O\;(2n^{5}log2n)$
	KNN	$O\;(n^{3}logn)$	$O\;(n^{4}logn)$	$O\;(n^{6}logn)$	$O\;(2n^{6}logn)$
	SDC	$O\;(n^{4}logn)$	$O\;(n^{5}logn)$	$O\;(n^{7}logn)$	$O\;(2n^{7}logn)$
	BLDC	$O\;(n^{3}logn)$	$O\;(n^{4}logn)$	$O\;(n^{6}logn)$	$O\;(2n^{6}logn)$
	MLP	$O\;(n^{6}logn)$	$O\;(n^{7}logn)$	$O\;(n^{9}logn)$	$O\;(2n^{9}logn)$
ResNet-152	SVM	$O\;({2n}^{4}logn)$	$O\;({2n}^{5}logn)$	$O\;({2n}^{7}logn)$	$O\;({4n}^{7}logn)$
	DT	$O\;(n^{2}log2n)$	$O\;(n^{3}log2n)$	$O\;(n^{5}log2n)$	$O\;({2n}^{5}log2n)$
	RF	$O\;(n^{3}log2n)$	$O\;(n^{4}log2n)$	$O\;(n^{6}log2n)$	$O\;(2n^{6}log2n)$
	KNN	$O\;(n^{4}logn)$	$O\;(n^{5}logn)$	$O\;(n^{7}logn)$	$O\;(2n^{7}logn)$
	SDC	$O\;(n^{5}logn)$	$O\;(n^{6}logn)$	$O\;(n^{8}logn)$	$O\;(2n^{8}logn)$
	BLDC	$O\;(n^{4}logn)$	$O\;(n^{5}logn)$	$O\;(n^{7}logn)$	$O\;(2n^{7}logn)$
	MLP	$O\;(n^{7}logn)$	$O\;(n^{8}logn)$	$O\;(n^{10}logn)$	$O\;(2n^{10}logn)$
DWAFF— ResNet-X	SVM	$O\;({4n}^{4}logn)$	$O\;({4n}^{5}logn)$	$O\;({4n}^{7}logn)$	$O\;({8n}^{7}logn)$
	DT	$O\;({2n}^{2}log2n)$	$O\;({2n}^{3}log2n)$	$O\;({2n}^{5}log2n)$	$O\;({4n}^{5}log2n)$
	RF	$O\;({2n}^{3}log2n)$	$O\;({2n}^{4}log2n)$	$O\;({2n}^{6}log2n)$	$O\;({4n}^{6}log2n)$
	KNN	$O\;({2n}^{4}logn)$	$O\;({2n}^{5}logn)$	$O\;({2n}^{7}logn)$	$O\;({4n}^{7}logn)$
	SDC	$O\;({2n}^{5}logn)$	$O\;({2n}^{6}logn)$	$O\;({2n}^{8}logn)$	$O\;({4n}^{8}logn)$
	BLDC	$O\;({2n}^{4}logn)$	$O\;({2n}^{5}logn)$	$O\;({2n}^{7}logn)$	$O\;({4n}^{7}logn)$
	MLP	$O\;({2n}^{7}logn)$	$O\;({2n}^{8}logn)$	$O\;({2n}^{10}logn)$	$O\;({4n}^{10}logn)$

## Data Availability

The data that support the findings of this study are available from the authors upon reasonable request.
